# The Present Status of Dental Service in the United States Army, and the Effect of War Conditions upon Dental Education

**Published:** 1918-11

**Authors:** R. Ottolengui


### THE PRESENT STATUS OF DENTAL SERVICE IN THE UNITED STATES ARMY, AND THE EFFECT OF WAR CONDITIONS UPON DENTAL EDUCATION

BY R. OTTOLENGUI

The increase of the draft age to forty-five has brought about a most embarrassing possibility for a large number of practicing dentists, viz.: the possibility of being drafted into the ranks. We have been inundated with letters asking such questions as the following: "Do you think it just for me, with fifteen years of practical experience, to be placed in the ranks, while men fresh from college have commissions?" "Are we being treated fairly? Have you heard of any physicians being drafted as foot soldiers?" "Is it not absurd to limit the dental service to one dentist per thousand men, when they have many times that number of doctors? Is it not a fact that 90 per cent. of the soldiers need dental attention, whereas not over ten or fifteen per cent. are really sick enough to require a doctor?" "I am willing to give up my practice, worth fifteen thousand dollars a year, but how can I

support my family on a foot soldier's pay?'' And from every point of the compass: ''What is the matter with the National Dental Association? Why does not our Legislative Committee introduce a bill increasing the number of dentists in the Army?''

It is not possible to take the time to reply to all such questions separately. Hence, authoritative information as to the present status of the Army Dental Service has been secured, and while the following statements have not the value of official utterance, they may be relied upon as absolutely accurate, and official dissemination of similar news will shortly occur.

In general answer to some of the questions it may be stated that many of those now anxious for commissions might have secured them had application been made a year ago. It is consequently, their own fault, or misfortune as the case may be, that at present they find nearly six thousand dentists ahead of them in the line. (No military jest intended.)

In regard to introducing a bill in Congress to increase the number of dentists in the Army, there are many vital reasons why it would have been unwise to do this. The public welfare will not permit us to be more specific. The desired increase has now been accomplished in another and far better manner. By which is meant, better for the soldiers, whose interests are paramount. The existing status is as follows:

What has been known as the Army Dental Reserve Corps, by a recent act has been discontinued. Hereafter all dentists serving in the U. S. Army will be known as members of the ''Dental Corps, U. S. A.''

At this date (September 13, 1918) 5,981 dentists hold commissions. Three thousand five hundred officers are already on duty.

Authorization has been secured for the commissioning and assignment of between 9,000 and 10,000 dentists before July 1, 1919.

This will require the commissioning of at least 3,000 dentists by July 1, 1919, and it is not impossible this number may be increased to 5,000.

Commissions, however, will not be open to civilian applicants until the dentists enlisted in the Army have had opportunity to be thus advanced. The following course will be pursued.

Examinations for dentists desiring commissions in the Dental Corps of the United States Army will be opened in the immediate future, first for such dentists as are now or may hereafter be acting as privates in various camps in this country and with troops overseas. Commissions will then be granted to the successful candidates as vacancies exist for the assignment of additional dental officers for the strength of the Army on that date.

In reference to civilian dentists who are at home and who desire commissions in the Dental Corps and are of conscription age and registered on September 12, 1918, no authority exists at this time for granting commissions for men who have not been inducted into military service. Should the regulation change and permit the commissioning of men, subject to draft, before being inducted into the military service, a decision will be reached upon commissioning such conscripts.

The only positive statement that can be made in reference to this question at this time, is, that so long as we have conscripted dentists in camps acting as privates, examinations will not be opened for dentists of conscription age who are at home. Vacancies existing for the assignment of dental officers will first be filled from among those who now hold commissions and desire immediate active duty. Second, from those men who are acting as privates and who secure commissions.

In case that more dentists should be inducted into the Army than can be given commissions there would be further opportunity to use them as dentists under the following regulation:

If the number of enlisted men exceed the number required as dental officers, four will be assigned as dental mechanics; three for the camp organization and one with each hospital located in the United States.

This will make it possible to rapidly construct all the partial dentures needed.

Dental assistants are authorized in the same grades and percentages within the grades as allowed for the enlisted men of the Medical Department up to and including the grade of Sergeant of the First Class.

All dental schools which were designated as "well recognized" in the Surgeon General's office will be permitted to make application for the establishment of the Students' Army Training Corps Unit for men between eighteen and twenty-one years of age who desire to study dentistry, and each school will be advised as to the number it will be permitted to enroll.

The decision has not yet been reached by the War Department as to whether or not students who are enlisted in the Medical Enlisted Reserve Corps will be immediately transferred to the Students' Army Training Corps, but said decision will be soon forthcoming.

A Military Director will be placed in charge at each dental school.

All students in the Students' Army Training Corps will be furnished all clothing without expense, and tuition will be paid by the Government, each man to receive pay of $30.00 a month. Quarters, consisting of barracks or suitable buildings that can be transformed to the purpose intended, will be constructed or rented by the school officials and approved by the Government's representative, and it is intended to house such students in a single group of buildings or barracks. All study and recreation hours will be under military supervision. Each school must secure drill grounds in close proximity, as these students must receive not less than six hours a week of military drill.

Examinations will be given every three months in military and professional subjects, and those who fall below an established grade will be dropped from the professional training and sent to camp for general military service. In so far as it is possible to do so, students in the Students' Army Training Corps will live under exactly the same military discipline as all other enlisted men live in the various camps.

It is assumed that there will be no vacation period for the dental students belonging to the Students' Army Training Corps. However, it is possible that the professional instruction may not be continuous, as all such students may be taken to camps for more extensive general military training during a portion of the usual summer vacation period. Still, it may be found necessary, in spite of this intensive training, to consume more than thirty-two teaching weeks to cover the normal professional course to make up for the time given over to military drill and military lectures.

Male students who are physically unfit for military service and foreign students who desire to do so, may purchase their own uniforms and receive the military training without additional expense beyond that referred to. Army cots, blankets and rifles will be furnished by the Government and sent to each school.—*Dental Items of Interest* for October.

